# Spring Zooplankton Community Structure and Its Relationship with Water Quality Gradients in Representative Nearshore Systems of the Beibu Gulf, China

**DOI:** 10.3390/ani16121847

**Published:** 2026-06-15

**Authors:** Shengjie Wang, Mingben Xu, Jun Zeng, Wengang Xu, Zhuheng Li, Shelei Li, Vin Cent Tai

**Affiliations:** 1 Faculty of Engineering, Built Environment, and Information Technology, SEGi University, Petaling Jaya 47810, Selangor, Malaysia; 15075630480@163.com (S.W.); taivincent@segi.edu.my (V.T.); 2 Guangxi Academy of Marine Sciences, Guangxi Academy of Sciences, Nanning 530007, China; xmben0771@163.com; 3 School of Ocean, Yantai University, Yantai 264005, China; 4 Beibu Gulf Marine Industry Research Institute, Fangchenggang 538000, China; 15878003705@163.com; 5 School of Information & Intelligence Engineering, University of Sanya, Sanya 572022, China; sheleili@sanyau.edu.cn

**Keywords:** community structure, eutrophication, Maowei Sea, Qinzhou Bay, Beilun Estuary, structural equation modeling, *Parvocalanus crassirostris*

## Abstract

This study integrated two surveys—one conducted in August 2021 in the Maowei Sea–Beilun Estuary (14 stations) and another in spring 2023 in Qinzhou Bay (16 stations)—to delineate four functional zones (MWS, BLE, WJ, WW). We systematically analysed zooplankton communities and water quality factors and used Structural Equation Modelling SEM to break down the effects of eutrophication on zooplankton into direct and indirect pathways. The results showed that sixteen zooplankton species belonging to six major taxonomic groups were recorded. Copepoda dominated the community, accounting for 50% of the total recorded species. *Parvocalanus crassirostris* reached a dominance index (Y) of 0.54, higher than historical records (0.35–0.40), indicating increased community homogenization. Salinity increased from 2.0 to 29.6 psu, resulting in community homogenization, and DIN decreased from 1.282 to 0.19 mg/L. Ei averaged 13.49 (MWS) and 12.80 (BLE). Zooplankton biomass rose nearly 7-fold (125 to 880 mg/m^3^, *p* < 0.05). SEM revealed a direct inhibitory pathway from eutrophication (−0.36), an indirect pathway via phytoplankton food-quality degradation (−0.14), and one via community high evenness compression (−0.06), for a total of −0.56 (Bootstrap 95% CI significant). The heavy-metal total effect (−0.18) was much weaker. The SEM fit was excellent (CFI = 0.976, RMSEA = 0.038, R2 = 0.68). This study is the first to quantify dual-indicator management thresholds (salinity < 10 psu and DIN > 0.8 mg/L) for early risk warnings in nearshore aquaculture in this region.

## Simple Summary

This study combined two systematic investigations of the nearshore areas of Qinzhou Bay and the Beilun River estuary. We used Structural Equation Modelling (SEM) to quantify three pathways through which eutrophication affected planktonic biomass. We observed a clear gradient from the inner to the outer bay: salinity increased, nutrient levels dropped, and eutrophication decreased. Copepods dominated the zooplankton community, with *Parvocalanus crassirostris* as the leading species. Its dominance exceeded historical levels, showing increased community homogenization. We used SEM to break down the effects of eutrophication on zooplankton into three measurable pathways: direct inhibition, indirect effects due to reduced food quality, and indirect effects due to community homogenization. The combined total effect was −0.56, indicating that food chain degradation and direct toxicity worked together.

## 1. Introduction

Zooplankton play an important role in energy transfer between primary producers and higher trophic levels in the marine food web, and their community dynamics directly affect the natural food supply during the juvenile stage of nearshore economic fish, represented by the orange-spotted grouper *Epinephelus coioides* [1,2,3]. The orange-spotted grouper is an important economically farmed fish species in subtropical and tropical sea areas. Gastric content analysis shows that its larvae have a significant selective feeding preference for planktonic animals such as copepods [4]. Feeding experiments have also shown that copepod nauplii can significantly improve the feeding and survival rates of larvae [5], making them a representative species in nearshore food webs and fisheries ecology research. In semi-enclosed nearshore bays, eutrophication and heavy metal enrichment caused by terrestrial material input often profoundly reshape the structure of planktonic animal communities through changes in phytoplankton community composition and the induction of low-oxygen zones at the bottom [6,7,8]. The multi-path regulation mechanism of quantifying water quality gradients on planktonic animal communities is of great significance for the assessment of nearshore ecosystems and the management of fishery resources [9,10].

The nearshore waters of Qinzhou Bay and the Beilun River mouth form a complete inner- and outer-bay gradient system. Maowei Sea (the inner Bay of Qinzhou Bay) has long been subjected to continuous freshwater inputs and land-based pollution from the Qinjiang and Maoling Rivers, resulting in pronounced eutrophication. The Beilun River estuary is strongly affected by runoff dilution, and surface salinity can drop sharply below 2.0 psu in summer, with substantial agricultural nitrogen and phosphorus pollution. The neck and outer bay areas show a significant improvement in water quality due to enhanced water exchange [11,12].

Qinzhou Bay, as a typical semi-enclosed bay in the northern Gulf of Guangxi, bears multiple pressures from domestic and industrial emissions as well as high-density aquaculture activities, forming a significant environmental gradient of eutrophication and heavy metal pollution, and providing an ideal research site for studying the composite ecological effects of multiple water quality factors [8]. Furthermore, according to regional monitoring data, domestic sewage accounts for 35% to 38% of total nitrogen sources in the bay, aquaculture for 32% to 36%, and industrial wastewater for 8% to 12%. Aquaculture accounts for 30% to 33% of the total phosphorus sources, while domestic pollution sources account for 17% to 20% [13,14]. The contribution rate of industrial sources to sediments ranges from 38% to 49%, making it the primary anthropogenic input. The urban runoff, aquaculture, and atmospheric deposition also provide varying degrees of replenishment [15,16]. Therefore, this gradient pattern from land to sea enables simultaneous investigation of acidification, salinity dilution, and heavy metal recombination within a single geographic unit.

Studies on nearshore zooplankton in the Beibu Gulf mainly focus on descriptive analysis of community composition [17,18], with few studies using structural equation modeling (SEM) to systematically quantify the direct effects of eutrophication on zooplankton and the indirect effect pathways transmitted through the planktonic community and high evenness under the premise of controlling for multivariate collinearity [19]. Chou et al. [20] found that river inputs mainly indirectly affect the diversity of planktonic animals through changes in phytoplankton. Zhang et al. [21] established a nutrient–primary productivity–zooplankton pathway model in Nanwan. Li et al. [22] found that both salinity and dissolved inorganic nitrogen have a strong impact on the biomass of planktonic organisms. However, there is still limited research on incorporating nutrient status, sediment element load, and community high evenness into the SEM framework in subtropical estuarine gradient systems. Therefore, this study investigated the plankton community, phytoplankton community, and water quality factors in the nearshore waters of Qinzhou Bay in the spring of 2023. Combined with the survey data from the Maowei Sea Beilun Estuary in August 2021, correlation analysis and SEM were used to quantify the direct and indirect effects of various water quality factors on the zooplankton community, aiming to provide a scientific basis for ecosystem assessment and aquaculture management in nearshore waters.

Considering that eutrophication and heavy metal pollution may affect the planktonic animal community by altering the status and community structure of phytoplankton resources, this article further proposes the following hypotheses: (1) eutrophication and heavy metal pollution could indirectly affect the biomass of planktonic animals by affecting phytoplankton density; (2) the characteristics of zooplankton communities may be influenced by changing the high evenness of the community; and (3) at the same time, it may also have a direct effect on the biomass of planktonic animals. The above assumptions will be tested within the framework of structural equation modeling (SEM).

## 2. Materials and Methods

### 2.1. Study Area and Survey Stations

**Figure 1:**
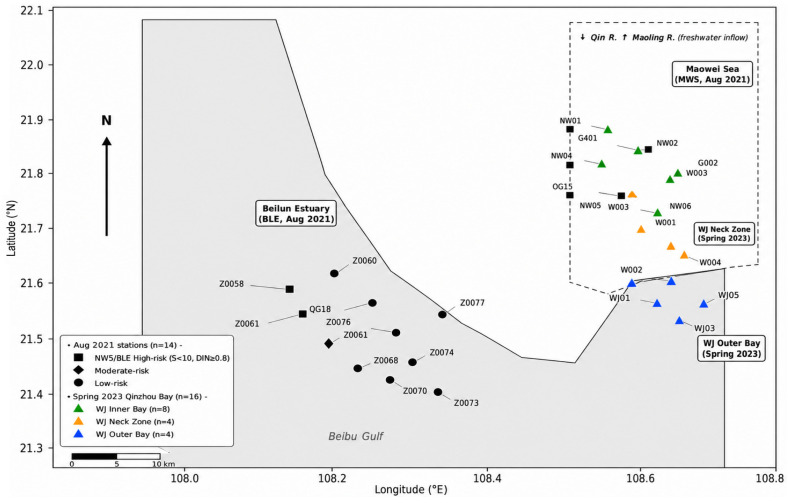
Study area and survey station distribution. The circles, diamonds and squares denote stations in August 2021. The triangles denote Qinzhou Bay stations in Spring 2023. MWS, Maowei Sea; BLE, Beilun Estuary; WJ, Neck Zone; WW, Outer Bay.

Sampling locations spanned Qinzhou Bay and the adjacent nearshore zones of Maowei Sea and Beilun Estuary (Figure 1). Four functional zones were divided based on hydrogeological characteristics, which were respectively as follows: (i) Maowei Sea (MWS), with 3 stations GX001, GX002 and Q035, surveyed in August 2021; (ii) Beilun Estuary (BLE)—eleven stations (ZQ059–ZQ080), also surveyed in August 2021; (iii) Neck Zone (WJ)—four stations (WJ01–WJ04) occupied in spring 2023, straddling the transitional boundary between inner- and outer-bay water masses; and (iv) Outer Bay (WW)—six stations (WW01–WW06).

In the spring of 2023, a total of 16 stations (6 in the NW district, 4 in the WJ district, and 6 in the WW district) were surveyed in Qinzhou Bay. Three parallel samples were collected at each station to ensure data reliability.

### 2.2. Surveys and Sample Collection

Fieldwork comprised two temporally and spatially separate campaigns that require strict distinction throughout. In August 2021, twenty-one stations were established across the Maowei Sea and the nearshore Beilun Estuary, and 14 complete station records were retained through quality control. Water quality, phytoplankton, chlorophyll a, and sediment samples were collected at each station.

In the spring of 2023, a systematic survey of zooplankton communities, phytoplankton communities, and water quality factors was conducted in Qinzhou Bay, covering three gradient sections, including the NW, WJ, and WW areas (*n* = 16 stations).

Sampling protocols followed the Chinese national oceanographic survey standards (GB/T 12763.4-2007; GB/T 12763.6-2007) [23,24] at all stations. Physicochemical measurements comprised salinity (SA), water temperature (WT), dissolved oxygen (DO), and eutrophication-related factors such as chemical oxygen demand (COD), dissolved inorganic nitrogen (DIN), and dissolved inorganic phosphate (DIP), as well as trace-metal analysis for Cu, Pb, Zn, Cr, Hg, Cd, and As.

Phytoplankton were filtered through a shallow-water type III plankton net and identified under an Olympus BX51 microscope. Zooplankton were captured with a shallow-water type II net. They were weighed on a balance after being fixed with 5% formaldehyde. The biomass was calculated in mg/m^3^. Shallow-water type II plankton nets (mesh size 160 μm, net diameter 37 cm) were used to vertically trawl zooplankton from the bottom to the surface at each station, and the filtered water volume was recorded by a flow meter (15–30 m^3^ per station). The samples were fixed with 5% formaldehyde on-site, and the biomass calculation was corrected according to the fixed shrinkage coefficient [18]. Phytoplankton were vertically trawled with a shallow-water type III net (mesh size 77 μm, net diameter 37 cm) and fixed with Lugol’s solution (final concentration 1%). Water quality samples (DIN, DIP, COD) were collected on-site and refrigerated at 4 °C before being transported back to the laboratory for pre-treatment within 24 h. The heavy metal samples were filtered through a 0.45 μm filter membrane and preserved by nitric acid acidification.

### 2.3. Data Analysis

#### 2.3.1. Biodiversity Indices

Community diversity was characterised using three complementary metrics. Shannon–Wiener diversity (***H***′), Pielou’s high evenness (***J***) and Margalef’s richness (***D***) were computed as follows:

***H***′ = −∑_***i***_ (***P***_***i***_ × ***log***_2_***P***_***i***_) (1)

***J*** = ***H***′/***log***_2_***S*** (2)

***D*** = (***S*** − **1**)/***log***_2_***N*** (3)

where ***S*** denotes total species richness, ***N*** the cumulative individual count, and ***P_**i**_*** = ***n_**i**_***/***N*** is the relative abundance of the ith taxon.

Dominance was indexed as

***Y*** = (***n**_**i**_*/***N***) × ***f**_**i**_* (4)

in which ***f_**i**_*** is the frequency of occurrence of species i across stations; taxa with ***Y*** ≥ 0.02 were designated dominant species [11].

#### 2.3.2. Eutrophication and Heavy-Metal Assessment

Eutrophication status was quantified via

***Ei*** = (***COD*** × ***DIN*** × ***DIP***)/(**4500** × **10**^−6^) (5)

where ***COD***, ***DIN***, and ***DIP*** were in mg/L. Waters with Ei ≥ 1 were classified as eutrophic [25]. The comprehensive heavy-metal water quality index (WQI) was calculated using a mean single-factor index:

***WQI*** = (**1**/***n***) × ∑ (***C_i_^s^***/***C_i_^n^***) (6)

where ***n*** = 7 metals (***Cu***, ***Pb***, ***Zn***, ***Cr***, ***Hg***, ***Cd***, ***As***), ***C_**i**_^s^*** was the measured aqueous concentration (μg/L) and ***C_i_^n^*** was the corresponding Class I seawater quality standard. Values exceeding unity (***WQI*** > 1) indicated composite Class I exceedance.

#### 2.3.3. Statistical Analyses

Inter-zone differences in water quality and biological indices were evaluated by one-way ANOVA (α = 0.05) with Duncan’s post hoc pairwise comparisons. Pearson product-moment correlations were calculated on the 16-station spring 2023 Qinzhou Bay dataset (*n* = 16; α = 0.05) to examine bivariate associations between water quality variables and zooplankton community metrics. Before analysis, the Shapiro–Wilk test was used to verify normality. The Levene’s test was used to verify homogeneity of variance, and all variables met the hypothesis without data conversion. Pearson correlation was used to confirm the linear relationship between variables through scatter plots.

#### 2.3.4. Structural Equation Modeling

**Figure 2:**
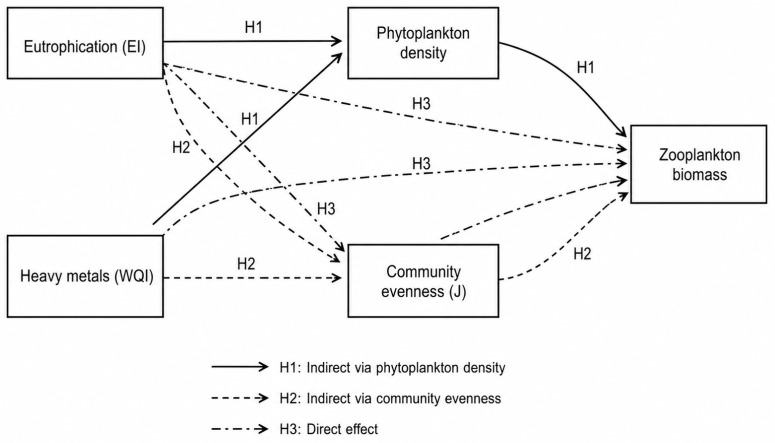
Hypothesised SEM path framework for water quality effects on zooplankton community structure. H1: indirect pathway via phytoplankton density; H2: indirect pathway via community high evenness; H3: direct pathway.

A structural equation model was specified with Ei and WQI as exogenous observed variables, phytoplankton density and high evenness J as latent mediators, and zooplankton biomass as the response (Figure 2). Three hypothetical pathways were encoded: H1 (eutrophication acting through phytoplankton density), H2 (eutrophication acting through community high evenness), and H3 (a direct eutrophication pathway).

Parameters were estimated by robust maximum likelihood (MLR), and indirect effects were obtained as products of standardised path coefficients:

***θ***_***indirect*** = ***β***_1_ × ***β***_2_ (7)

where ***β***_1_ was the exogenous-to-mediator coefficient and ***β***_2_ was the mediator-to-response coefficient. Significance of indirect effects was evaluated by bootstrapping (1000 resamples; 95% CI), with non-zero confidence intervals taken as evidence of significance. Acceptable model fit was defined by CFI ≥ 0.90, TLI ≥ 0.90, SRMR ≤ 0.08 and RMSEA ≤ 0.08. Computations were performed in R 4.5.1 using the lavaan package [26]. The observation indicators of each latent variable in the SEM measurement model were as follows: Eutrophication (Ei) was composed of the eutrophication index Ei, DIN, and DIP. The comprehensive pressure of heavy metals (WQI) was characterized by the comprehensive pollution index WQI of Cu, Pb, Zn, and Cr. The density of phytoplankton is directly observed as cell density (cells/L). The community high evenness was measured as the Pielou index J, and the biomass of Zooplankton was measured as wet weight (mg/m^3^). The standardized factor loadings of each latent variable were all greater than 0.50 [27]. Model fitting was evaluated using CFI (>0.90), RMSEA (<0.08), SRMR (<0.08), and χ^2^/df (<3.0) [28], and alternative models were compared using AIC. The final model was the one with significant path coefficients and the smallest AIC.

## 3. Results

### 3.1. Spatial Distribution of Water Quality Parameters

**Figure 3:**
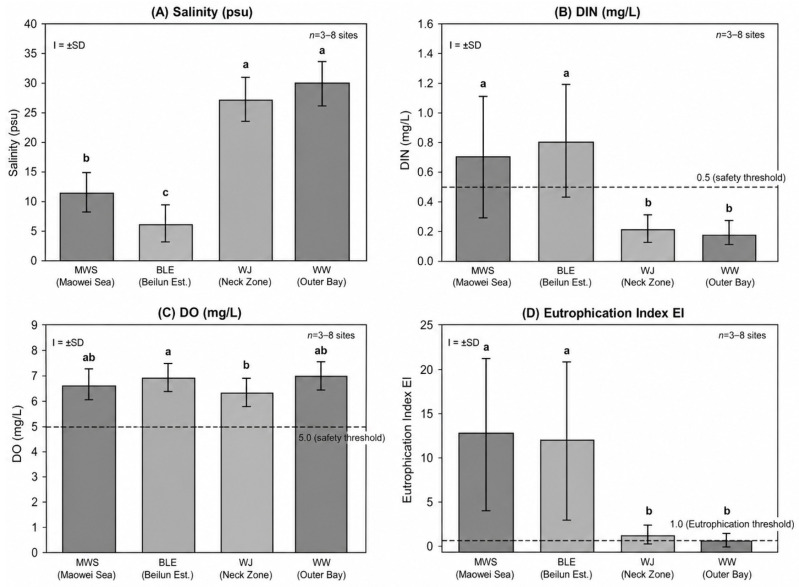
Water quality parameters across the four functional zones. Data were expressed as Means ± SD. The different letters indicate significant differences (*p* < 0.05).

Significant spatial differentiation in all measured water-quality variables was detected across the four zones (Table 1, Figure 3). Salinity gradually increased from BLE to WW. The BLE stations registered a minimum of 5.4 ± 3.8 psu. The MWS was 11.4 ± 3.6 psu, and WW was the highest at 29.6 ± 1.12 psu (*p* < 0.01). The DIN content was 3.8–4.3-fold higher in the MWS and BLE relative to the WW (*p* < 0.05), revealing a synergistic pattern of low salinity and high nutrition. The average dissolved oxygen (DO) in the surface layer was between 4.64 and 7.26 mg/L, and the surface DO at the ZQ076 station (4.64 mg/L) was close to the critical value for fish respiratory safety (5 mg/L) [7], indicating a potential low-oxygen risk.

Eutrophication indices (Ei) in MWS were 13.49 ± 10.61 and in BLE 12.80 ± 9.50, which were significantly higher than in WW (1.14 ± 1.27, *p* < 0.01). The MWS, BLE, and WJ were in a state of eutrophication, with the indicator Ei ≥ 1. The composite WQI showed no significant difference (*p* > 0.05).

Within the spring 2023 survey in Qinzhou Bay, significant physicochemical gradients from NW through WJ to WW were observed (Table 2). Mercury (Hg) at the inner-bay margin (NW) surpassed the Class I standard (0.05 μg/L) and was significantly higher than concentrations at WJ or WW (*p* < 0.01).

### 3.2. Phytoplankton Community and Chlorophyll a Distribution

**Figure 4:**
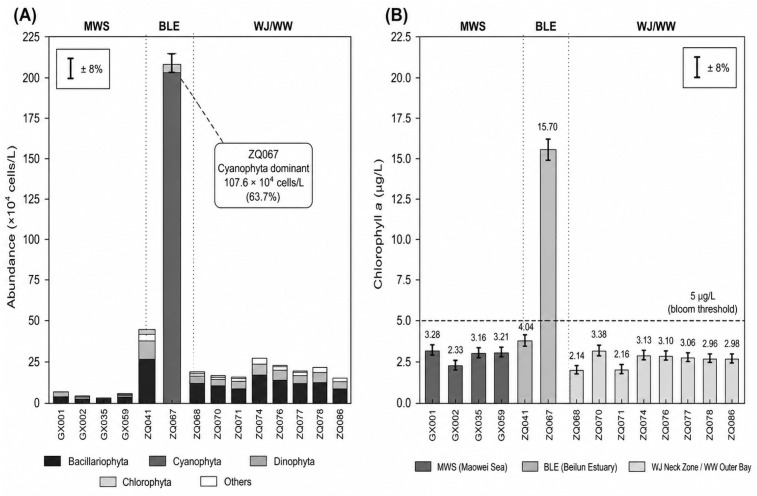
Phytoplankton community composition (**A**) and chlorophyll a concentration (**B**) at each station in the August 2021 Maowei Sea–Beilun Estuary survey (*n* = 14). Red dashed line: bloom threshold (5 μg/L). Vertical dotted lines in panel (**B**) mark the sampling station corresponding to the peak chlorophyll a value of 15.70 µg/L. Error bars: ±8%.

The total abundance range of phytoplankton was from 2.92 to 168.80 × 10^4^ cells/L, with Bacillariophyta as the dominant group at each station (Figure 4A). At BLE Station ZQ067, Cyanophyta showed a sharp increase and reached 107.60 × 10^4^ cells/L, accounting for 63.7% of the total phytoplankton. At the same time, DIN and COD were also at their peak at this station. Chlorophyll a was as high as 15.70 μg/L (Figure 4B), suggesting that the station has obvious risk characteristics of an algal bloom.

### 3.3. Composition and Dominant Species of Zooplankton Communities

During the investigation, a total of 16 species of zooplankton were recorded, belonging to six major categories (Table 3). Among them, copepods have the largest number of species, accounting for 50% of the total recorded species, and are the main component group of the surveyed marine plankton community. Planktonic larvae account for 18.75%, Chaetognatha species account for 12.5%, and other groups have relatively low proportions. The analysis of dominant species showed that *Parvocalanus crassirostris* was the first dominant species, with a dominance Y value of 0.54, greatly higher than that of other species. Balanus larvae and Copepod larvae are the second most dominant species, with dominance levels of 0.04 and 0.03, respectively. Overall, the surveyed marine zooplankton community exhibits a community structure dominated by copepods and a few dominant species.

### 3.4. Spatial Pattern of Indicators for Zooplankton Communities

**Figure 5:**
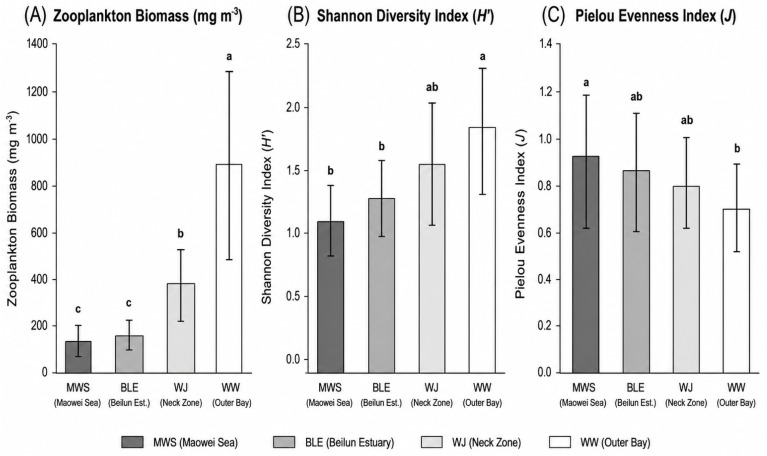
Zooplankton community metrics across the four functional zones. Data are presented as Mean ± SD. The different letters indicate significant differences (*p* < 0.05).

The zooplankton biomass increased from the MWS station to WW, and the WW and WJ were significantly higher than those in MWS and BLE (*p* < 0.05, Figure 5A). The Shannon–Wiener diversity index H′ also gradually increased, while there was no significant difference between them (*p* > 0.05, Figure 5B). However, Pielou’s high evenness J decreased in reverse, with the MWS station being significantly higher than the WW station (*p* < 0.05, Figure 5C).

### 3.5. Correlations Between Water Quality and Zooplankton Community Metrics

**Figure 6:**
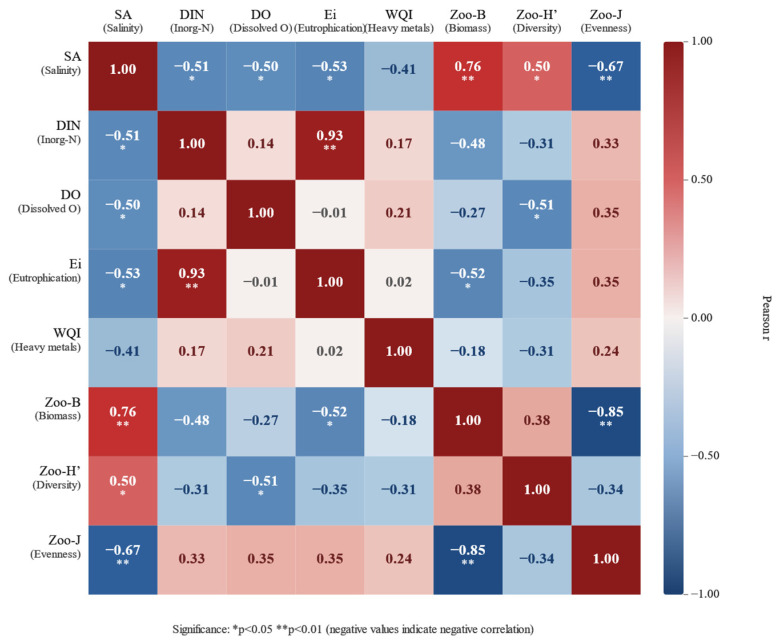
Pearson correlation coefficient matrix for the spring 2023 Qinzhou Bay survey (*n* = 16 stations). Blue: negative correlation; red: positive correlation.

The Pearson correlation analysis results present the response pattern of zooplankton biomass to water quality gradients (Figure 6). The zooplankton biomass is positively correlated with salinity (r = 0.66, *p* < 0.05), while it is significantly negatively correlated with the eutrophication index Ei (r = −0.73, *p* < 0.01) and high evenness J (r = −0.61, *p* < 0.05). It is negatively correlated with the WQI, but not at a significant level (r = −0.27, *p* > 0.05).

Among the water-quality variables, Ei was strongly positively correlated with DIN (r = 0.91, *p* < 0.01) and DIP (r = 0.88, *p* < 0.01), and had a strongly negative correlation with salinity (r = −0.87, *p* < 0.01). Pielou’s high evenness J was positively correlated with Ei (r = 0.64, *p* < 0.05), revealing the mechanistic characteristics of the inhibition of community homogenization in highly eutrophic areas.

### 3.6. Path Analysis of Structural Equation Modeling

**Figure 7:**
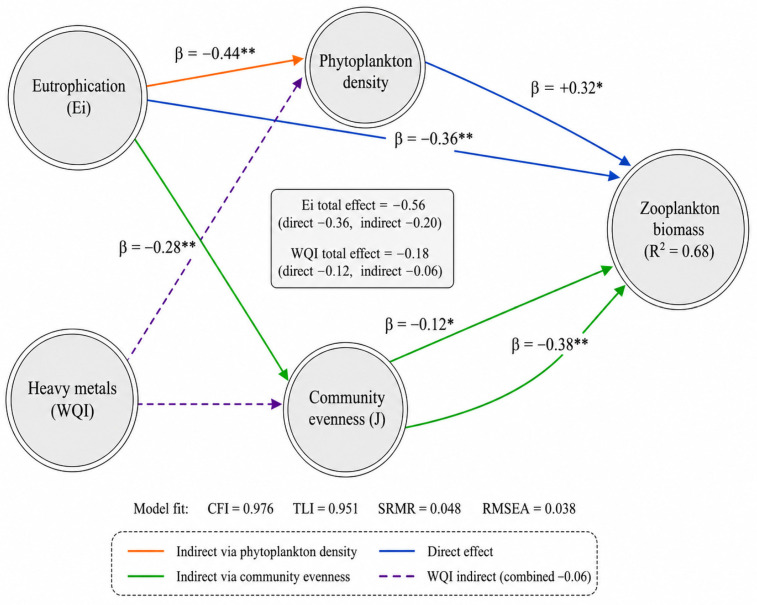
SEM results and effect decomposition. Standardised path coefficients shown on each arrow; Bootstrap 95% CI in parentheses. Note: H2 path β_1_ = +0.16 reflects a positive Ei–J relationship (high eutrophication drives higher evenness under stress). R^2^ = 0.68. Note: Path coefficients are standardized estimates. * indicates statistical significance at *p* < 0.05, and ** indicates statistical significance at *p* < 0.01.

Satisfactory convergence was achieved for the final SEM (CFI = 0.976, TLI = 0.951, SRMR = 0.048, RMSEA = 0.038; Figure 7). The standardized direct path coefficient of Ei on planktonic biomass was −0.36 (Bootstrap 95% CI: −0.51 to −0.21, *p* < 0.01).

In terms of indirect pathways, through phytoplankton density (H1), eutrophication exerted an indirect effect of −0.14 (β_1_ = −0.44, β_2_ = 0.32).

The community-high evenness indirect pathway (H2) yielded a smaller effect of −0.06 (β_1_ = 0.16, β_2_ = −0.38). The positive β_1_ coefficient captures the empirical pattern whereby higher Ei raises high evenness J—a pseudo-high evenness signal—which then suppresses biomass via negative β_2_.

Summing direct and both indirect effects gives a total Ei effect of −0.56 (indirect subtotal −0.20, R^2^ = 0.68). The WQI direct pathway coefficient was −0.12 (*p* < 0.05), and the total WQI effect was −0.18. The model explained 68% of the variance of planktonic biomass (R^2^ = 0.68), indicating excellent overall fitting quality.

## 4. Discussion

### 4.1. Community Characteristics of Zooplankton

In the present study, sixteen zooplankton species belonging to six major taxonomic groups were recorded, with copepods representing 50% of the total recorded species. Similar to previous investigations in Qinzhou Bay, copepods remained the dominant zooplankton group [11,17]. A seasonal survey conducted near the coast of the Beibu Gulf found that copepods remained the first dominant group throughout the year. The results in this study are highly consistent with these reports, suggesting the stability of copepods as the backbone group of zooplankton communities in subtropical estuaries and bays. *Parvocalanus crassirostris* is the most ecologically representative dominant species in the survey area of this study, with a dominance degree of 0.54, far exceeding the values 0.35–0.40 recorded by Pang et al. [11]. Compared to historical surveys, an increase in its dominance was observed, which may be related to changes in environmental conditions and community structure adjustments in recent years. However, its specific ecological adaptation mechanism still needs to be further verified through long-term monitoring data and experimental research. Lan et al. [18] also ranked high in the list of dominant species in the 2011–2012 Qinzhou Bay survey, showing good continuity with the dominant species pattern in this study. However, the magnitude difference deserves continuous monitoring.

### 4.2. Comparison of Biomass Spatial Patterns

In this study, the biomass of zooplankton showed a 7-fold gradient from the inner bay (125 mg/m^3^) to the outer bay (880 mg/m^3^). A survey conducted by Lan et al. [18] in Qinzhou Bay from 2011 to 2012 indicated that the biomass in the inner bay was lower than that in the outer bay. The spatial pattern of the results in this study was completely consistent with the reported results, indicating that this distribution pattern was lower in the inner bay and higher in the outer bay, and had temporal stability in Qinzhou Bay.

It is worth noting that the biomass of 880 mg/m^3^ in the outer bay in this study was higher than the data of 500–650 mg/m^3^, suggesting that the primary productivity or bait conditions in the outer bay may have improved and may also be affected by interannual climate variability. Furthermore, Pang et al. [17] conducted a seasonal survey near the coast of the Beibu Gulf and found that the overall biomass in summer was higher than that in spring. In this study, the spring mean was about 317 mg/m^3^, which is basically consistent with the recorded spring data. Song et al. [29] also reported a positive correlation between copepod biomass and salinity and a negative correlation with DIN in the coastal waters of the Greater Bay Area in Guangdong, Hong Kong, and Macao. The results in this study are consistent with the above reports, indicating that the regulation of nearshore copepod biomass by salinity and nutrient gradient is universal in nearshore bays of South China.

Yan et al. [9] found in their survey of the Beibu Gulf that the diversity of fishery resources in high-eutrophication areas near the coast was low, which may be related to the simplification of the food chain structure caused by the singularity of zooplankton communities. This is consistent with the ecological mechanism revealed by the high evenness and low biomass pattern in the inner bay in this study [30,31]. Xiao et al. [32] recorded a positive correlation between the biomass of zooplankton and the density of economic fish larvae and juveniles in the Yellow Sea. In this study, the biomass level in the inner bay was low, only 125–160 mg/m^3^, which may pose a natural bait constraint on the juvenile stage of farmed fish during the spring breeding season. Further quantitative evaluation is needed in conjunction with the survival rate of farmed fish [3,33].

### 4.3. Multi-Pathway Regulation Mechanism of Eutrophication

In this study, the total effect of eutrophication revealed by SEM was −0.56, which far exceeds that of heavy metal pollution at −0.18. Zhang et al. [34] reported that eutrophication is the first dominant water quality factor in the Yalu River during winter. The results of this study are consistent with the reported results in terms of factor priority. However, in this study, the overall effect magnitude was larger, which is speculated to be related to the suitable spring temperature and the more sensitive response of phytoplankton and copepods to nutrient changes [35,36]. Sun et al. [37] used multiple regression analysis in Jiaozhou Bay to find that nutrient enrichment restricts the biomass of copepods, which is consistent with the direction of this study. Furthermore, this study divided the mechanism into three pathways through SEM, providing a more detailed analysis.

The ecological meanings of the three pathways were different. In the direct inhibition pathway (−0.36), eutrophication had a direct toxic effect on zooplankton by deteriorating the physical and chemical environment of water bodies, such as low oxygen and high ammonia nitrogen. The effect of ZQ059 with low dissolved oxygen indicates a potential threat of bottom layer hypoxia in the inner bay [7], which is consistent with the report by Pang et al. [11] regarding the overall low population of zooplankton in Qinzhou Bay during spring. In the indirect pathways mediated by phytoplankton density (−0.14), eutrophication did not increase the supply of high-nutrient-value phytoplankton, but instead promoted the evolution of the community towards the dominance of cyanobacteria. The abundance of cyanobacteria at station ZQ067 appeared simultaneously with high DIN, high COD, and high chlorophyll-a, which is a typical example of this transformation. There is experimental evidence for the inhibitory effect of blue-green algae toxins on the feeding and reproduction of copepods [6], which is consistent with the food web degradation pattern observed by Zheng et al. [38] in eutrophic bays of the Mediterranean.

In the indirect pathway mediated by high evenness (−0.06), under high eutrophication conditions in the inner bay, the biomass of all species is suppressed with high evenness, rather than the succession path of specific small species with single expansion reported in the Xiangshan Port model [39]. The differences may be due to regional differences in adaptation strategies of dominant species and water exchange intensity. In a healthy community, a small number of dominant species contribute the majority of total biomass, and high evenness is relatively low [40]. Under high eutrophication, dominant species are selectively suppressed due to food quality degradation and cyanobacterial toxicity, leading to more equitable species abundances and a passive increase in high evenness while total biomass simultaneously declines [6]. This passive homogenization is consistent with the dominance effect described by Hillebrand et al. [41] that ecosystem biomass is closely tied to dominant species, and their loss leads to net biomass decline even when high evenness increases. This pattern is also supported by observations from Lan et al. [18] in the inner bay of Qinzhou Bay during the wet season. Furthermore, Lan et al. [18] also observed a pattern of higher high evenness of zooplankton communities in the inner bay than in the outer bay during the wet season of Qinzhou Bay, which is consistent with the results of this study, indicating that this mechanism of homogenization inhibition is representative in Qinzhou Bay.

### 4.4. Suggestions for Nearshore Ecological Management

Based on the quantitative results of this study, the following suggestions are proposed for the management of nearshore ecosystems in the research area. Firstly, eutrophication prevention and control should be made the primary goal of ecological management in the inner bay. Focusing on the Qinjiang and Maoling River basins, we will promote the reduction in agricultural non-point source nitrogen and phosphorus emissions, gradually reduce the total DIN load in the Inner Bay, and control Ei below the eutrophication threshold (Ei = 1) [42,43,44].

Secondly, a dual indicator warning mechanism should be established for the flood season, with salinity below 10 psu and DIN above 0.8 mg/L as the trigger thresholds for warning. During the high incidence period of typhoons and heavy rainfall from July to September, regular preventive reminders will be issued to farmers, and measures such as reducing breeding density and emergency oxygenation will be taken in advance.

Thirdly, implement zoning management of aquaculture carrying capacity: the water quality in the Outer Bay (WW area) was good (Ei < 2), and the aquaculture carrying space was relatively sufficient. During the flood season, high-risk areas near the Neiwan (NM) and Beilun river mouths should actively reduce the density of net cages and minimize the aquaculture losses caused by multiple stressors [45,46].

Finally, continuous long-term monitoring of mercury traceability should be carried out. The mercury concentration in the inner bay (0.06 μg/L) was three times that of the outer bay. Although it has not yet exceeded the first-class seawater quality standard, the risk of methylmercury biomagnification in the food chain could not be ignored [47].

### 4.5. Limitations of This Study

This study analyzed the data from two surveys conducted in the Maowei Sea and the Beilun River estuary area in August 2021 and the Qinzhou Bay area in spring 2023. Because the survey times and regions were not completely consistent, the differences between functional areas may be influenced not only by spatial environmental factors but also by seasonal changes. Therefore, the results of this study mainly reflect the ecological characteristics of each surveyed sea area during the survey period, and caution is still needed when interpreting the spatial distribution pattern.

Furthermore, due to the limitations of the survey conditions, the number of stations in each functional area is relatively limited, and the reflection of local-scale environmental heterogeneity may be insufficient. The structural equation model is based on existing ecological knowledge, but some potential influencing factors, such as the biological effects of different forms of heavy metals and the role of hydrodynamic processes, have not yet been included in the analysis framework, and their impact still needs further research.

During the investigation, the appearance of cyanobacteria was recorded, but the identification work was mainly based on conventional microscopic observations, only reaching the level of classification, and no taxonomic identification was carried out at the genus or species level. At the same time, this study did not conduct detection of cyanobacterial toxins, so it is impossible to determine the composition and ecological risks of potential toxin-producing cyanobacteria. Further research can be conducted on the structure and ecological effects of cyanobacterial communities by combining molecular biology methods and cyanobacterial toxin monitoring. At the same time, strengthening continuous investigations in different seasons, combined with stable isotope and hydrodynamic simulation analysis, will help to provide a more comprehensive understanding of the changes in plankton communities and their driving mechanisms in the nearshore waters of Guangxi [48,49].

## 5. Conclusions

This study synthesized two systematic investigations of the nearshore areas of Qinzhou Bay and the Beilun River estuary and, for the first time, quantitatively analyzed the three mechanistic pathways of eutrophication affecting planktonic biomass in the sea area using SEM. We found the following:

(1) A great gradient of increasing salinity, decreasing nutrient content, and decreasing eutrophication degree has formed in the research area from the inner bay to the outer bay. The Ei of Maowei Sea and Beilun River estuary both far exceeded the eutrophication threshold. Independent survey data of Qinzhou Bay in spring 2023 confirm the stability of this pattern.
(2) The zooplankton community was mainly composed of copepods, with *Parvocalanus crassirostris* as the absolute dominant species. The dominance degree was higher than historical records, indicating an increasing trend of community homogenization. The biomass increased in a gradient of about 7 times from Maowei Sea to the outer bay, with a reverse change in evenness, suggesting that eutrophication was inhibiting zooplankton in the inner bay.
(3) This study innovatively uses SEM to decompose the impact of eutrophication on zooplankton into three quantifiable pathways: direct inhibition, the indirect pathway of food quality deterioration, and the indirect pathway of community homogenization. The total effect of these pathways was −0.56, revealing the synergistic effect of the dual mechanisms of food chain quality degradation and direct physiological toxicity.

This study proposes, for the first time, an operable dual-index flood season warning threshold (salinity < 10 psu and DIN > 0.8 mg/L) for the sea area, providing a scientific basis for quantitative early warning of nearshore net-cage fish farming risks. It is recommended to prioritize the prevention and control of eutrophication in the inner bay for nearshore ecological management, promote non-point source emission reduction in the Qinjiang and Maoling River basins, reasonably regulate the spatial layout of aquaculture, and carry out multi-year, four-season systematic monitoring to support the health of nearshore food webs and the sustainable development of aquaculture.

## Figures and Tables

**Table 1:** Water quality parameters and pollution risk indices across the four functional zones.

Zone	SA (psu)	DIN (mg/L)	DO (mg/L)	Ei	WQI	n
MWS (Maowei Sea)	11.4 ± 3.6 ^c^	0.72 ± 0.37 ^a^	6.5 ± 0.40 ^ab^	13.49 ± 10.61 ^a^	1.21 ± 1.02 ^a^	3
BLE (Beilun Est.)	5.4 ± 3.8 ^d^	0.82 ± 0.41 ^a^	6.9 ± 0.50 ^a^	12.80 ± 9.50 ^a^	1.05 ± 0.90 ^a^	11
WJ (Neck Zone)	27.5 ± 1.37 ^b^	0.23 ± 0.04 ^b^	6.33 ± 0.28 ^b^	2.28 ± 0.63 ^b^	0.61 ± 0.54 ^a^	4
WW (Outer Bay)	29.6 ± 1.12 ^a^	0.19 ± 0.06 ^b^	6.99 ± 0.34 ^ab^	1.14 ± 1.27 ^b^	0.95 ± 0.88 ^a^	6
F-value	68.5 **	9.1 **	4.2 *	6.3 **	0.82	—

Note: Data were expressed as Means ± SD. The different letters indicated significant differences (*p* < 0.05). The MWS and BLE data were from August 2021. The WJ and WW data were from Spring 2023. SA, salinity; DIN, dissolved inorganic nitrogen; DO, dissolved oxygen; Ei, Eutrophication indices; WQI, comprehensive heavy-metal water quality index; n, station number. * *p* < 0.05; ** *p* < 0.01.

**Table 2:** Survey data from the spring 2023 Qinzhou Bay survey across three functional zones (Mean ± SD).

	SA (psu)	DO (mg/L)	COD (mg/L)	DIN (mg/L)	DIP (mg/L)	Ei	WQI	Mercury (μg/L)
NW (Inner)	14.66 ± 2.55 ^b^	7.16 ± 0.62 ^a^	2.14 ± 0.60 ^a^	0.72 ± 0.37 ^a^	0.04 ± 0.02 ^a^	13.49 ± 10.61 ^a^	1.21 ± 1.02 ^a^	0.06 ± 0.02 ^a^
WJ (Neck)	27.45 ± 1.37 ^a^	6.33 ± 0.28 ^b^	1.54 ± 0.27 ^ab^	0.23 ± 0.04 ^b^	0.03 ± 0.01 ^ab^	2.28 ± 0.63 ^b^	0.61 ± 0.54 ^a^	0.03 ± 0.02 ^b^
WW (Outer)	29.61 ± 1.12 ^a^	6.99 ± 0.34 ^ab^	1.14 ± 0.42 ^b^	0.19 ± 0.06 ^b^	0.02 ± 0.01 ^b^	1.14 ± 1.27 ^b^	0.95 ± 0.88 ^a^	0.02 ± 0.01 ^b^
F-value	110.02 **	4.44 *	6.72 **	8.32 **	4.97 **	5.15 **	0.79	9.34 **

Note: * *p* < 0.05; ** *p* < 0.01. The different lowercase letters in the same column indicate significant differences.

**Table 3:** Taxonomic composition and dominance index of zooplankton recorded during the survey.

Group	Scientific Name	Dominance Index (Y)
Chaetognatha	*Sagitta bedoti*	—
Chaetognatha	*Flaccisagitta enflata*	—
Tunicata	*Oikopleura dioica*	—
Cladocera	*Penilia avirostris*	—
Copepoda	*Euterpina acutifrons*	—
Copepoda	*Parvocalanus crassirostris*	0.54
Copepoda	*Paracalanus parvus*	—
Copepoda	*Oithona similis*	—
Copepoda	*Corycaeus affinis*	—
Copepoda	*Temora discaudata*	—
Copepoda	*Acartia pacifica*	—
Copepoda	*Microsetella norvegica*	—
Polychaeta	*Trochophore*	—
Planktonic larvae	*Balanus larva*	0.04
Planktonic larvae	*Copepod larva*	0.03
Planktonic larvae	*Brachyura zoea*	—

Note: The symbol ‘—‘ indicates that the dominant species standard has not been met.

## Data Availability

The data supporting the findings of this study are available from the corresponding authors upon reasonable request.

## Abbreviations

The following abbreviations are used in this manuscript:

BLE	Beilun Estuary
Chl a	Chlorophyll a
CI	Confidence interval
COD	Chemical oxygen demand
DIN	Dissolved inorganic nitrogen
DIP	Dissolved inorganic phosphorus
DO	Dissolved oxygen
Ei	Eutrophication index
MWS	Maowei Sea
NW	Inner-bay margin zone
RMSEA	Root mean square error of approximation
SA	Salinity
SEM	Structural equation modeling
SRMR	Standardised root mean square residual
TLI	Tucker–Lewis index
WJ	Neck Zone
WQI	Water quality index
WW	Outer Bay
WT	Water temperature
CFI	Comparative fit index
